# Supination resistance variations in foot and ankle musculoskeletal disorders: implications for diagnosis and customised interventions with wedged insoles

**DOI:** 10.1186/s13047-023-00681-5

**Published:** 2023-12-21

**Authors:** Gabriel Moisan, Dominic Chicoine, Sean McBride, Nader Farahpour, Pier-Luc Isabelle, Camille Dagenais, Ian Griffiths

**Affiliations:** 1 Department of Human Kinetics, Université du Québec à Trois-Rivières, Trois-Rivieres, Canada; 2 Groupe de recherche sur les affections neuro-musculo-squelettiques (GRAN), Université du Québec à Trois-Rivières, Trois-Rivieres, Canada; 3 Department of Physical Therapy, University of Findlay, Findlay, OH USA; 4 Department of Sport Biomechanics, Faculty of Sport Sciences, Bu Ali Sina University, Hamedan, Iran; 5 Sports and Exercise Medicine, William Harvey Research Institute, Queen Mary University of London, London, UK

**Keywords:** Foot, Ankle, Musculoskeletal diseases, Physical examination, Biomechanical phenomena

## Abstract

**Background:**

Supination resistance is a clinical outcome that estimates the amount of external force required to supinate the foot. A greater supination resistance may indicate greater loads on structures responsible for generating internal supination moments across the subtalar joint during static and dynamic tasks. As such, greater supination resistance may be an expected finding in medial foot and ankle musculoskeletal disorders, such as plantar fasciopathy (PF) and posterior tibial tendon dysfunction (PTTD), whereas reduced supination resistance may be present in lateral ankle disorders, such as chronic ankle instability (CAI). However, no studies have yet investigated the changes in supination resistance across these foot and ankle musculoskeletal disorders. This study aimed to quantify supination resistance in individuals with PF, PTTD and CAI compared to healthy controls. Additionally, this study aimed to explore the changes in supination resistance following the simulation of varus and valgus wedges, which are commonly used interventions for these disorders.

**Methods:**

Fourteen participants with PF, fourteen with PTTD, fourteen with CAI and fourteen healthy controls were recruited. Supination resistance was quantified on a level surface and on a 10-degree inclined surface with varus and valgus positions.

**Results:**

Supination resistance was lower for the injured foot for CAI (*p* < 0.001) and greater for PTTD (*p* < 0.001) compared to the healthy foot. There was no significant between-foot difference observed for PF (*p* = 0.275) and controls (*p* = 0.970). In the injured foot, CAI exhibited lower supination resistance compared to controls (*p* < 0.001), PF (*p* = 0.012) and PTTD (*p* = 0.014). Regardless of the groups, supination resistance increased when tested on a surface with valgus inclination (*p* < 0.001) and decreased when tested on a surface with varus inclination (*p* < 0.001).

**Conclusions:**

Varus and valgus inclinations to the surface were effective in modifying supination resistance in PTTD and CAI, respectively. Supination resistance seemed unchanged in PF, and thus inclining the standing surface leads to greater between-feet asymmetries. This study also highlights the potential of wedged insoles as a mean to customise treatments and modify tissue stresses in these disorders. The findings contribute to the understanding of foot and ankle biomechanics and may aid in the development of more effective management and rehabilitation strategies.

## Background

Musculoskeletal disorders affecting the foot and ankle are prevalent within both the general population and athletes [1, 2]. Among these disorders, plantar fasciopathy (PF) [3], chronic ankle instability (CAI) [4], and posterior tibial tendon dysfunction (PTTD) [5] stand out as some of the most common and debilitating ailments. Plantar fasciopathy is a degenerative and overuse condition that primarily affects the insertion of the plantar aponeurosis at the medial calcaneal tubercle [6]. Clinically, this disorder presents with tenderness at the inferomedial calcaneus, and patients report intense pain during the first few steps after a period of rest [6]. Additionally, a positive windlass test, during which passive dorsiflexion of the first metatarsophalangeal joint reproduces heel pain [7], underscores the link between the disorder and tensile loading of the plantar fascia late in the stance phase, to raise the arch and supinate the foot [8]. Chronic ankle instability (CAI) is characterised by a propensity for recurrent lateral ankle sprains at least 12 months after the initial sprain, with frequent episodes of the ankle “giving way.” Persistent symptoms include pain, swelling, limited range of motion, weakness, and diminished self-reported function [9]. Mechanically, CAI is associated with increased ankle inversion [10], a laterally deviated center of pressure [11], greater peak pressures under the lateral forefoot [12], and increased peroneus longus activity [10, 11] during gait. Interventions, such as wedged insoles and foot orthoses, targeting these alterations have been suggested in the literature [13]. Posterior tibial tendon dysfunction (PTTD) encompasses various underlying causes, with the primary cause being excessive tensile forces that strain the posterior tibial tendon. These forces may lead to chronic inflammation and structural degeneration, and changes in the composition and organisation of collagen bundles within the tendon structure [14–16]. Ultimately, PTTD may result in acquired flatfoot deformity over the course of several stages [14–16]. Pain, impaired function, and decreased quality of life are associated with all three of these disorders [17–19].

Despite an extensive volume of literature directed towards various interventions for these disorders, the effectiveness of current treatments still requires significant improvement [20–24]. Additionally, the literature suggests that these disorders are at least partly related to tissue loading, which may make the traditional clinical examination suboptimal since few clinical examination techniques assess the loading/forces (kinetics) of the weightbearing foot. A tissue stress approach, proposed by McPoil and Hunt [25], offers a framework for the examination and treatment of foot and ankle disorders. According to this approach, the assessment aims to identify and address excessive tissue loads, and treatments are specifically targeted towards modifying tissue stresses. A recent randomised controlled trial supported this approach for the treatment of PF [26]. However, the exact relationships between most clinical tests and dynamic tissue loads remain largely unknown. One test that may provide insight into the kinetics of the foot and ankle is the supination resistance test (SRT) [27]. Recent studies have demonstrated that the SRT, when performed using a handheld device, is reliable [28] and is related to midfoot kinetics during gait [29].

Historically, the SRT has been used to understand the amount of vertical lifting force required to supinate the weightbearing foot. It has been postulated that greater resistance to supination may indicate greater loads on structures responsible for pronation control or producing supination moments across the subtalar joint (STJ) during static and dynamic tasks [27]. Considering the substantial variability found in the anatomical location of the STJ axis between individuals [30], it is reasonable to anticipate a significant diversity in tissue stresses as a result. Payne et al. [31] found a moderate correlation (*r* = 0.59) between the SRT and the alignment of the subtalar joint axis. Essentially, the findings of Payne et al. [31] suggest that a more medially deviated STJ axis is associated with greater resistance to supination, whereas a more laterally deviated STJ axis is associated with decreased supination resistance [31]. From a clinical perspective, conditions such as PF and PTTD have been associated with feet exhibiting medially deviated STJ axes [32]. This deviation can result in excessive stresses on tissues responsible for resisting pronation and facilitating supination [32]. Conversely, it has been suggested that a more laterally deviated STJ axis may be correlated with CAI [32]. According to the subtalar joint axis location and rotational equilibrium theory of foot function (SALRE) [32], individuals with PTTD or PF would be expected to demonstrate greater supination resistance. In contrast, less supination resistance would be expected for those with CAI. If patients with different musculoskeletal disorders demonstrate variations in supination resistance, the clinical utilisation of SRT could potentially aid developing rehabilitation plans that incorporate the mitigation of tissue stresses. This approach may contribute to more effective injury management and rehabilitation strategies.

The use of wedged insoles has been identified as an intervention capable of altering tissue stresses in individuals with PF [33], PTTD [34], and CAI [35]. According to SALRE and the tissue stress approach [25, 32], these devices apply external forces that can reduce the load on injured structures. Valgus wedges provide external pronatory moments across the STJ, and thus should increase supination resistance. Conversely, varus wedges provide external supinatory moments, and therefore should decrease supination resistance. However, there are no published data available to validate these hypotheses. Enhancing our comprehension of the impact of wedges on supination resistance carries significant potential for clinicians and researchers, enabling them to target treatments for individuals with foot and ankle musculoskeletal disorders.

The objectives of this study were two-fold: Firstly, to determine if supination resistance differs across musculoskeletal disorders (CAI, PF, PTTD) compared to controls and to identify the differences in supination resistance between injured and healthy feet. Secondly, to investigate the changes in supination resistance observed in individuals with musculoskeletal disorders after simulating varus and valgus wedging. It was hypothesised that individuals with CAI would exhibit the lowest supination resistance, followed by controls, PF and PTTD. The injured foot was expected to exhibit a significant difference compared to the healthy foot across all groups (except the control group). The simulated inclined surfaces were expected to attenuate the differences in supination resistance, bringing the values closer to those of the healthy foot across all groups.

## Methods

### Participants

The sample size was calculated using G Power 3.1.9.7 (Heinrich Heine University, Düsseldorf, Germany). As no previously published studies compared supination resistance across individuals with musculoskeletal disorders or investigated the effects of surface inclination on supination resistance, the sample size was determined using the pilot data of the first 34 recruited participants of this study. A sample size between 24 and 56 participants was determined to be adequate to obtain a power of 80%, considering alpha of 0.05 and partial Eta Squared of 0.053 and 0.133 for the supination resistance comparisons between feet and between groups on a level surface, respectively.

Fourteen participants with PF, 14 with CAI, 14 with PTTD and 14 healthy controls were recruited between April 2022 and August 2023 to participate in this multicenter case-control study (Level III Evidence). Participants were recruited from the outpatient podiatry clinic located at Université du Québec à Trois-Rivières (UQTR) in Trois-Rivières, Canada, and the PodFormance outpatient podiatry clinic in Québec City, Canada. Additionally, recruitment efforts were extended through email and social media invitations.

To be included in the study, all participants needed to be at least 18 years old. The inclusion criteria for the PF group were self-reporting heel pain with a minimum intensity of 3 (on a scale from 0 to 10) during walking or palpation of the plantar fascia insertion on the calcaneum, and experiencing symptom aggravation during the initial steps after a period of rest (i.e., post-static dyskinesia) occurring at least five times per month. The inclusion criteria for the CAI group were established based on the recommendations of the International Ankle Consortium [36] and comprised: having a history of at least one ankle sprain that was sustained 12 months prior to the participation in the study, and the presence of symptoms such as the ankle “giving way,” recurrent sprains, and/or a perception of ankle instability. The inclusion criteria for the PTTD group were experiencing pain located at the medial ankle or foot, pain elicited upon palpation of the posterior tibial tendon, a positive double and/or single heel-rise test, and being diagnosed with a stage 1 or 2 PTTD according to Johnson and Strom’s classification [14]. The exclusion criteria for all groups were as follows: experiencing a lower limb musculoskeletal disorder other than the specific pathologies under investigation within 3-month prior to data collection, having a history of a lower limb orthopedic surgery, and being diagnosed with a neuromuscular disorder that could alter balance control or foot and ankle morphology (e.g., multiple sclerosis, Parkinson’s disease, stroke).

The study protocol was approved by the UQTR Ethic Committee (CER-22-285-07.04). All participants provided their written consent prior to the experimentation.

### Protocol

Demographic and anthropometric data, consisting of age, sex, body mass, height, and foot posture assessed with the Foot Posture Index (FPI-6) was recorded and the Foot and Ankle Ability Measure-Activities of daily living subscale (FAAM-ADL) and Foot and Ankle Ability Measure-Sports subscale (FAAM-S) questionnaires were administered to all participants. Additionally, for the CAI group, the number of sustained lateral ankle sprains, frequency of episodes of instability, time since the first and last sprains and the score of the Cumberland Ankle Instability Tool (CAIT) were recorded. For the PTTD group, the PTTD stage according to Johnson and Strom’s classification was also collected. Demographic data are presented in Table 1.

**Table 1:** Demographic data. Data are displayed as mean(SD) unless specified otherwise

Group	Control	PF	CAI	PTTD
**Gender ratio (M/F)**	6/8	4/10	2/12	1/13
**Age (year)**	42.9 ($\pm$ 18.7)	50.0 ($\pm$17.4)	27.3 ($\pm 5.0$)	$55.0 (\pm$19.1)
**Body mass (kg)**	68.6 ($\pm$ 13.5)	80.4 ($\pm 7.3$)	71.5 ($\pm$ 12.9)	79.7 ( $\pm$ 14.2)
**Height (cm)**	167.1 ($\pm 7.9$)	168.3 ($\pm$11.3)	167.3 ($\pm$ 10.0)	161.0 ($\pm$ 7.4)
**Injured side or dominant side (R/L)**	12/2	8/6	13/6	4/10
**FPI-6 injured foot**	4.8 ($\pm$ 3.4)	3.6 ($\pm 2.8)$	6.0 ($\pm 2.7$)	9.3 ($\pm$ 2.2)
**FPI-6 healthy foot**	4.6 ($\pm$ 3.3)	3.4 ($\pm2.4)$	6.6 ($\pm$ 3.0)	6.4 ($\pm$ 2.8)
**FAAM ADL (/84)**	84.0 ($\pm 0.0)$	63.2 ($\pm$ 15.3)	75.3 ($\pm$6.7)	55.4 ($\pm$ 17.6)
**FAAM S (/32)**	32.0 ($\pm 0.0)$	18.7 ($\pm$ 10.6)	23.6 ($\pm$ 5.9)	13.2 ($\pm$ 6.9)
**Time since last sprain (months)**	-	-	38.8 ($\pm$ 38.1)	-
**Time since first sprain (months)**	-	-	146.5 ($\pm$ 80.6)	-
**Number of sustained sprains**	-	-	5.7 ($\pm$ 3.3)	-
**Give way frequency (nb/month)**	-	-	4.2 ($\pm$ 3.5)	-
**CAIT (/30)**	-	-	17.1 ($\pm$ 4.7)	-
**Duration of symptoms (months)**	-	6.6 ($\pm$ 4.9)	-	8.2 ($\pm$ 7.1)
**Johnson and Strom Classification (stage I/stage II)**	-	-	-	4/10

#### Supination resistance test

**Fig. 1:**
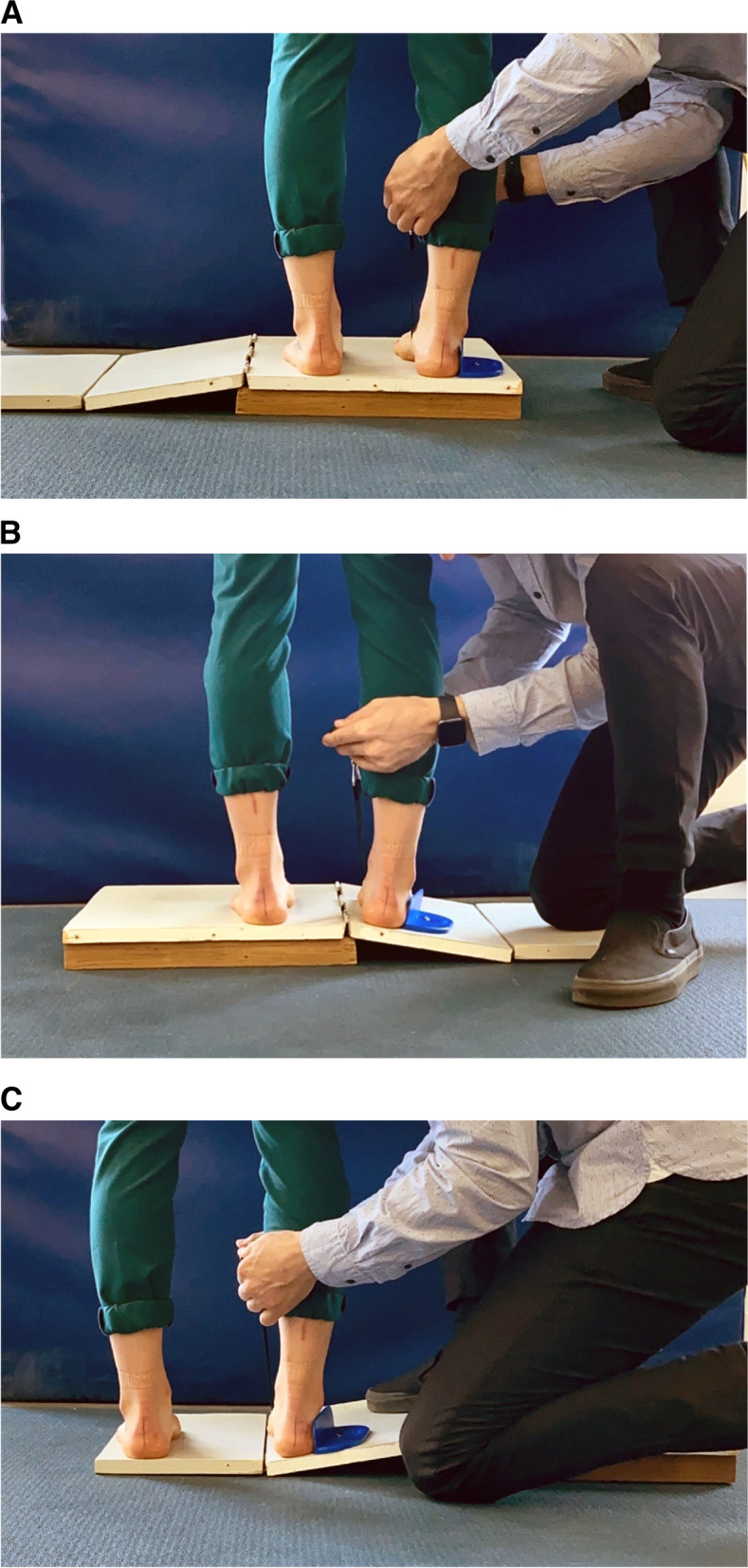
**a** SRT on the level surface. **b** SRT on the 10-degree varus inclined surface. **c** SRT on the 10-degree valgus inclined surface

 Supination resistance data were collected by a licensed podiatrist, who is a member of the teaching staff of the podiatric medicine program at UQTR, possessing six years of clinical experience. The validated method, previously described by Moisan et al. [28], was used to measure supination resistance with the Keystone device. First, the evaluator drew a vertical bisection line on the posterior aspect of the participant’s calcaneum to serve as a visual reference for the hindfoot inversion during the test. Participants were asked to perform five steps in place, to adopt a natural standing position, look straight ahead and put equal weight on both feet. Next, the non-stretchable 25 mm wide strap, was passed under the foot from the calcaneocuboid joint to the medial posterior part of the navicular tuberosity. The anchor was placed on the lateral side of the foot while the force gauge of the Keystone device was held on the medial side of the foot. The evaluator then applied a vertical traction to the device to create a hindfoot inversion movement at a consistent speed. Once the value of the vertical traction force remained stable, the device was automatically locked and the value was recorded as the final measurement. Whilst pulling, the evaluator was blinded to the measure by a paper screen attached to the Keystone Device to ensure that he was not biased by the values during the execution of the SRT. The participants were also asked not to help nor resist against the force applied on their foot during the data collection. The supination resistance data were collected on a three-piece wooden platform. This platform comprised an elevated level surface, a 10-degree inclined surface, and a second level platform, all connected with metal hinges (see Fig. 1a). Five supination resistance measures were taken for each foot on the elevated level surface. On the 10-degree inclined surface, five measures were taken while in inversion (varus inclination position) for the injured foot (see Fig. 1b), and an additional five measures were taken while in eversion (valgus inclination position) (see Fig. 1c). The order of the tasks was randomised across participants using a random table number to avoid a sequence bias.

**Table 2:** Normalised age-adjusted mean supination resistance across groups and surfaces. Data are displayed as mean [95% confidence intervals]

Group	Control	PF	CAI	PTTD
Mean SRT Healthy (%BW)	14.0 [12.8–15.2]	12.6 [11.5–13.7]	13.1 [10.8–15.4]	10.4 [9.3–11.5]
Mean SRT Injured (%BW)	14.0 [12.9–15.2]	13.0 [12.0-14.1]	8.9 [6.8–11.0]	13.0 [12.0-14.1]
Mean SRT Varus (%BW)	11.6 [10.5–12.6]	11.0 [10.0–12.0]	8.8 [6.9–10.8]	10.1 [9.1–11.0]
Mean SRT Valgus (%BW)	16.5 [15.3–17.7]	14.6 [13.5–15.7]	12.1 [9.8–14.4]	14.6 [13.5–15.7]

### Statistical analysis

Data were analysed with SPSS-28.0.1.0 (IBM Corporation, Armonk, NY, USA). Initially, the normality of the distribution of the demographic and supination resistance data was evaluated using visual (histograms/probability graphs) and analytical (Kolmogorov–Smirnov/Shapiro–Wilk test) methods. As all data were normally distributed, one-way ANOVAs with Tukey’s post-hoc tests were used to compare descriptive variables between the groups. Then, a repeated measure ANOVA with one within-subject factor (foot) with two levels (injured foot & healthy foot), one between-subject factor (group) with four levels (control, PF, CAI, and PTTD), and a covariate (age) was used. The main effects of the foot factor, and the group factor, as well as the interaction between the « foot » and « group » factors, were calculated. To compare supination resistance on all surfaces across all groups for the injured foot (dominant for controls), a repeated measures ANOVA with one within-subject factor (surface) with three levels (medially inclined, level and laterally inclined), one between-subject factor (Group) with four levels (Control, PF, CAI and PTTD), and a covariate (age) was used. Pairwise comparisons were used as post-hoc tests. Age-adjusted mean supination resistance values are reported. The significance level was set at 0.050 for all analyses and Bonferroni corrections were applied to adjust for multiple comparisons.

## Results

### Demographic data

**Fig. 2:**
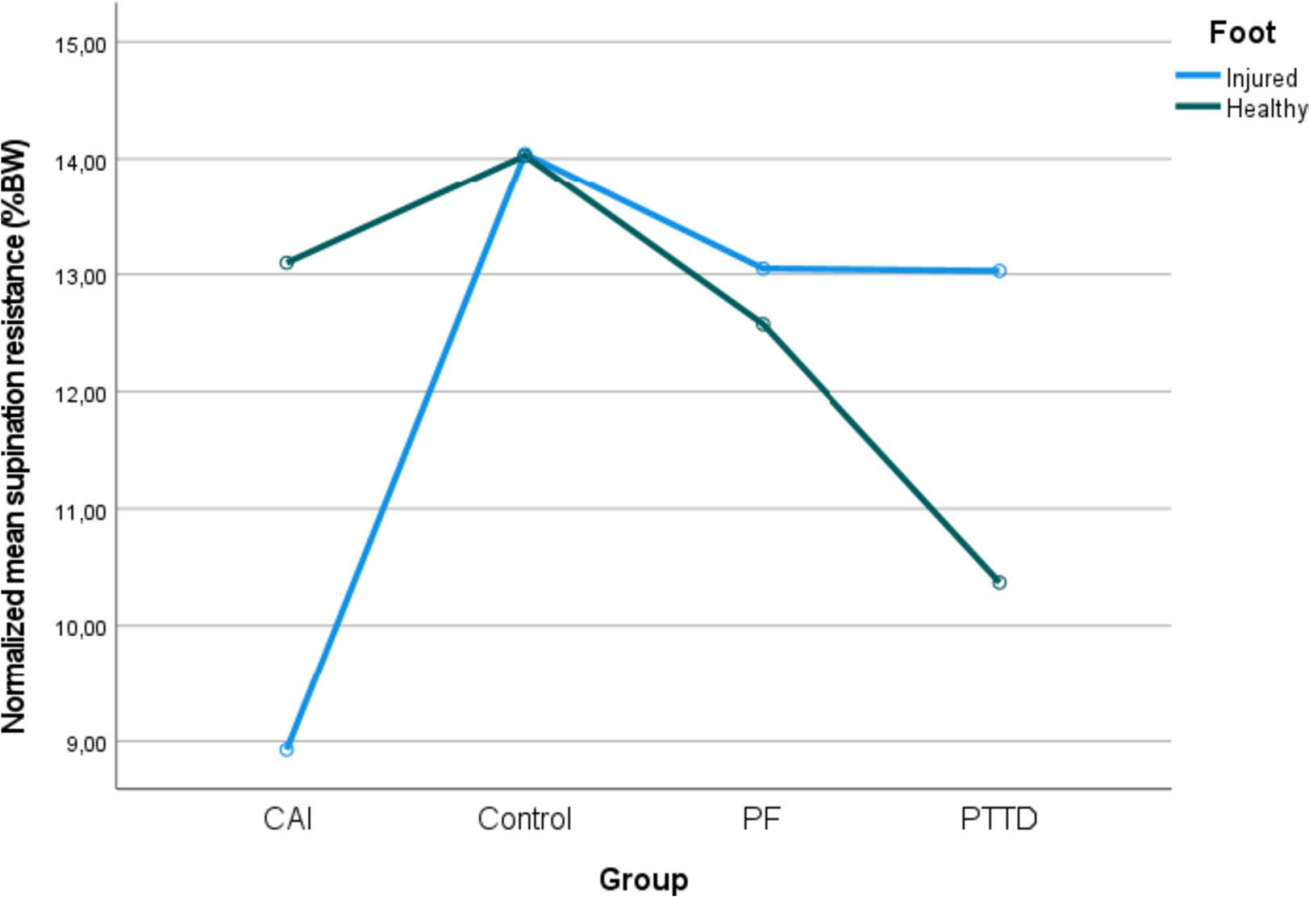
Normalised age-adjusted mean supination resistance of the injured and healthy foot

**Fig. 3:**
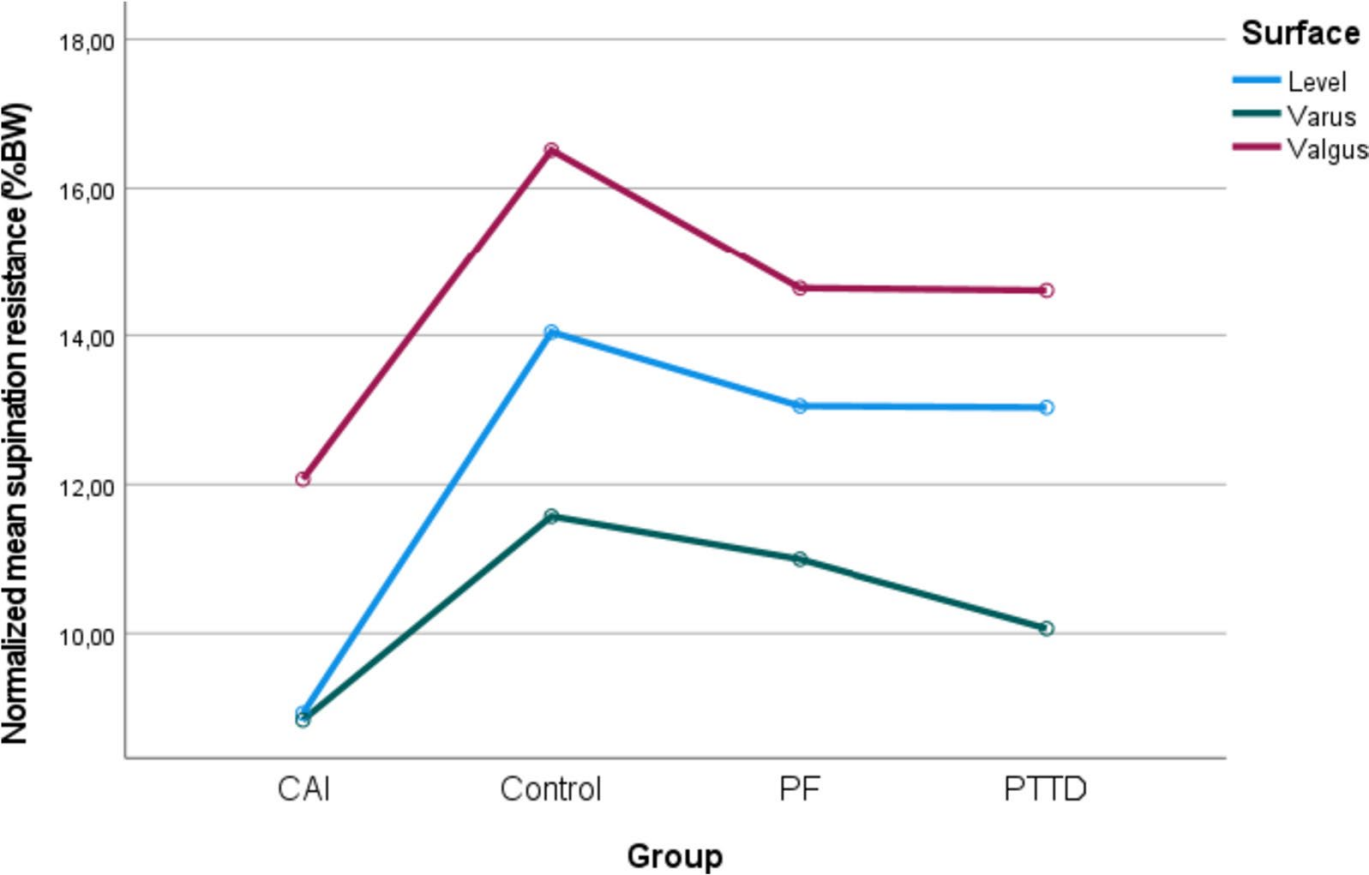
Normalised age-adjusted mean supination resistance during level, varus and valgus inclinations

 Even though the ANOVA detected a significant difference in body mass across groups (*p* = 0.028), the post-hoc tests revealed no significant difference (*p* > 0.050). There were no significant between-group differences for height (*p* = 0.156). Mean age was lower for CAI compared to PF (*p* = 0.003) and PTTD (*p* < 0.001). Mean FPI-6 was greater for PTTD compared to controls (*p* < 0.001), PF (*p* < 0.001) and CAI (*p* = 0.016). Mean FAAM-ADL was lower for PTTD compared to CAI (*p* < 0.001) and controls (*p* < 0.001), for PF compared to controls (*p* < 0.001) and CAI (*p* = 0.043). Mean FAAM-S was greater for controls compared to PF (*p* < 0.001), CAI (*p* = 0.013) and PTTD (*p* < 0.001) and for CAI compared to PTTD (*p* = 0.003). Demographic data are presented in Table 1. Supination resistance data are presented in Table 2; Figs. 2 and 3.

### Between-foot differences during the level surface condition

There was a significant Foot X Group interaction (*p* < 0.001). Supination resistance was lower for the injured foot for CAI (*p* < 0.001) and greater for PTTD (*p* < 0.001) compared to the healthy foot. There was no between-foot difference for PF (*p* = 0.275) and controls (*p* = 0.970).

### Between-group and between-condition differences for injured/dominant feet

There was significant Surface (*p* < 0.001) and Group effects (*p* = 0.003) as well as Surface X Group interactions (*p* < 0.001). Regardless of surfaces, CAI exhibited lower supination resistance than controls (*p* = 0.009). Regardless of groups, supination resistance during valgus inclination was greater than during level (*p* < 0.001) and compared to varus inclination (*p* < 0.001). Supination resistance during valgus inclination was also greater than during level inclination (*p* < 0.001). However, for CAI, there was no significant difference between supination resistance during level and varus surface (*p* = 1.000).

During level surface, CAI exhibited lower supination resistance than controls (*p* < 0.001), PF (*p* = 0.012) and PTTD (*p* = 0.014). During valgus inclination, CAI exhibited lower supination resistance than controls (*p* = 0.012). During varus inclination, there were no significant differences across groups (*p* > 0.050).

## Discussion

This study aimed to investigate variations in supination resistance among individuals with different musculoskeletal disorders (CAI, PF, PTTD) compared to controls, identify differences in supination resistance between injured and healthy feet for each group, and understand how changes in supination resistance observed in individuals with musculoskeletal disorders can be modified by simulating varus and valgus wedges.

Consistent with our hypothesis, individuals with CAI exhibited lower supination resistance in the injured foot compared to the healthy foot. Chronic ankle instability is characterised by greater ankle joint mobility and ligamentous laxity due to damage sustained by the lateral ankle ligaments [37]. The lateral ligaments of the ankle play a crucial role in providing stability and enhancing joint stiffness during the ankle joint complex supination [9]. Therefore, when these ligaments are weakened, stretched, or torn, it is likely to contribute to lower supination resistance. The 32% and 36% reductions in supination resistance for the injured foot in CAI compared to the healthy foot and the dominant foot of the control group are worrisome (see Table 2; Fig. 2). Individuals with CAI frequently experience functional limitations that predispose them to the ankle “giving way”, putting them at a higher risk of recurrent ankle sprains [9, 38]. Sustaining multiple lateral ankle sprains can lead to the development of long-term joint degenerative sequelae, such as post-traumatic ankle osteoarthritis [39]. Additionally, this may lead to a decrease in physical activity levels [40] and a decline in health-related quality of life [41]. Considering that individuals with CAI present deficits in proprioception [42], delayed activation [43], and weakness of the evertor muscles [44], they often encounter difficulties in preventing excessive movement when the ankle joint complex begins to invert. As a result, they are more prone to ankle sprains. The decreased supination resistance further contributes to this cascade considering that less force is required to induce the supination of the ankle joint complex, increasing the vulnerability to injuries.

In accordance with our hypothesis, individuals with PTTD exhibited greater supination resistance to their injured foot compared to their healthy foot. Posterior tibial tendon dysfunction is primarily caused by the excessive tensile forces that overload the posterior tibial tendon [45]. The posterior tibial muscle is the main invertor of the ankle joint complex and supinator of the STJ [14, 15, 46]. Failure of the posterior tibial tendon to support the foot can lead to ligamentous lesions to the medial ankle ligaments, such as the spring ligament, which then contributes to loss of stability [47], and increased rigidity of the foot and ankle as the pathology progresses [14, 15]. These morphological changes likely contribute to greater supination resistance, as observed in our study. Furthermore, it was previously hypothesised that PTTD is associated with a medial deviation of the subtalar joint axis [32] which is correlated with greater supination resistance [31]. However, our study does not allow us to determine the association between subtalar joint axis location and supination resistance in PTTD and thus further investigations are warranted. Contrary to our hypothesis, PTTD did not present greater supination resistance than controls and there were no significant differences in supination resistance between the injured and healthy feet in PF.

Moreover, the supination resistance in PF was not significantly different from the healthy counterparts. Plantar fasciopathy is characterised by microtears in a thickened plantar fascia [48], but unlike PTTD and CAI, PF does not usually lead to ligamentous or muscles injuries [48, 49]. It appears that one important factor that could influence supination resistance is foot and ankle mobility/rigidity and that the specific structures affected in PF do not have a direct impact on supination resistance. PTTD is associated with greater foot and ankle rigidity [14, 15], while CAI is characterised by enhanced mobility of the ankle joint complex [9]. In contrast, PF does not exhibit alterations in arch stiffness [50] which may explain the absence of changes in supination resistance.

In agreement with our hypothesis, regardless of the groups, the supination resistance was significantly increased during valgus inclination and decreased during varus inclination; it increased by 18% and decreased by 15%, respectively. The only exception was CAI, for which there was no significant difference in supination resistance between level and varus inclinations. This result could perhaps be explained by a reflex activation of the evertor muscles (e.g., peroneus longus and brevis) as a preventive mechanism to avoid excessive inversion of the rearfoot. However, as muscle activity of the lower limb was not investigated, further studies are needed to validate this hypothesis. For CAI, the valgus inclination increased the supination resistance to 12.1%BW, which brought the value closer to that of the healthy feet (from 32 to 8% between-foot difference). Similarly, the varus inclination decreased the supination resistance to 10.1%BW in PTTD, which brought the value closer to that of the healthy feet (from 25 to 3% between-foot differences) (see Fig. 3). In the case of PF, no differences in supination resistance were found between the injured and healthy feet. It is important to note that the varus and valgus inclinations introduced significant asymmetries between feet when compared to the healthy foot on the level surface. These asymmetries could potentially have detrimental effects in individuals with PF.

### Clinical and research perspectives

Addressing the lower supination resistance in CAI and the greater supination resistance in PTTD during the rehabilitation process may be beneficial. Long-term, this approach may help alleviate symptoms, prevent recurrent injuries, reduce long-term joint degeneration, and enhance the physical activity level and quality of life for patients with CAI and PTTD. Foot orthoses or insoles with varus or valgus posting/wedging may be beneficial in patients with PTTD and CAI, respectively. Clinical trials investigating this hypothesis are warranted.

Even though this study allowed us to quantify the differences in supination resistance between three foot and ankle musculoskeletal disorders, the cross-sectional design does not allow to determine whether the observed differences are a cause or a consequence of each disorder. A prospective study investigating the differences in supination resistance before and after developing CAI and PTTD is needed to determine whether the supination resistance changes are a cause or a consequence of these musculoskeletal disorders.

Also, previous results suggested that the measures of the supination resistance test are strongly correlated with foot and ankle kinetics during walking [29]. However, it is still unknown whether between-group and between-foot differences in supination resistance during standing are correlated with changes in foot and ankle biomechanics during locomotion. Thus, further studies are warranted to clarify this. Further studies are needed to investigate whether greater changes in supination resistance when inclining the standing surface (e.g., with wedges) are correlated with greater biomechanical alterations during locomotion. This will allow developing predictors of biomechanical effects of external aids (i.e., wedged insoles and foot orthoses) with the overarching goal of improving function and attenuate pain in those with musculoskeletal disorders.

### Limitations

Firstly, the mean age was significantly different between the groups, which could be explained by the disorders we investigated. In order to facilitate recruitment, a decision was made not to match the mean age across groups. However, to ensure that the age of the participants did not bias the results, ANCOVAs with age as a covariate were used. Secondly, a wood platform was used to medially and laterally incline the surface with the objective to simulate wedged insoles. However, the platform was not entirely representative of insoles. For example, patients often wear wedged insoles under both feet. In our study, the platform only inclined the tested foot which could have changed weight distribution compared to insoles worn under both feet. Clinically, wedging is often only placed under the rearfoot rather than the entire length of the foot and an angle of 10^o^ is not commonly used. We suggest being cautious when extrapolating the results of our study to insole conditions. Thirdly, our study allowed us to determine that inclining the standing surface is effective in modifying supination resistance values. However, the supination resistance test evaluates individuals in a static position which may perhaps not be entirely representative of the forces generated by medial ankle structures during dynamic tasks. Fourthly, plantar fasciopathy was diagnosed using clinical measurements. Using ultrasound imaging would have increased the validity of the diagnosis and allowed to evaluate plantar fascia thickening.

## Conclusions

PTTD exhibited greater supination resistance in the injured foot compared to the contralateral healthy foot. CAI exhibited a lower supination resistance in the injured foot compared to controls and PF, as well as compared to the healthy contralateral foot. Varus and valgus inclinations to the surface were effective in modifying supination resistance in PTTD and CAI, respectively. Supination resistance seemed unchanged in PF, and thus inclining the standing surface leads to greater between-feet asymmetries.

## Data Availability

The datasets used and/or analysed during the current study are available from the corresponding author on reasonable request.

## Abbreviations

%BW: Percentage of the body weight
CAI: Chronic ankle instability
CAIT: Cumberland Ankle Instability Tool
FAAM-ADL: Foot and Ankle Ability Measure-Activities of daily living subscale
FAAM-S: Foot and Ankle Ability Measure-Sports subscale
FPI-6: Foot Posture Index
PF: Plantar fasciopathy
PTTD: Posterior tibial tendon dysfunction
SRT: Supination resistance test
STJ: Subtalar joint axis
UQTR: Université du Québec à Trois-Rivières
